# Supplementary material for: Seroprevalence Study of Conserved Enterotoxigenic Escherichia coli Antigens in Globally Diverse Populations
Source: Microorganisms. 2023 Aug 31;11(9):2221. doi: 10.3390/microorganisms11092221 (PMC10536235; doi:10.3390/microorganisms11092221)
Supplement: Supplementary file 1 [file microorganisms-11-02221-s001.zip › microorganisms-2514156-supplementary.pdf]

Supplementary materials

Supplemental Methods

ELISA development and optimization with sera from vaccinated mice

All samples used in ELISA development and optimization were collected during prior experiments and no new animal experiments were performed for this study. In brief, these studies used sera from CD1 mice previously vaccinated with purified recombinant EatA passenger domain (rEatAp), recombinant EtpA (rEtpA), or LT through intramuscular, intradermal, or orogastric routes and subsequently challenged with ETEC [1,2]. Sera from these prior experiments was collected and stored at 4°C for future use. All prior animal experiments were performed under protocols approved by the Animal Studies Committee of Washington University School of Medicine (20110246A1) and procedures complied with Public Health Service guidelines and the guide for the care and use of Laboratory animals.

ELISA plates were prepared as described in the methods section. To further optimize the ELISA conditions, checkerboard assays were used to determine secondary antibody dilutions that maximized the signal-noise ratio. We determined that secondary antibody dilutions of 1:20,000 were optimal for both anti-mouse IgG (Invitrogen 62-6720) and anti-mouse IgA (cell signaling technology, 7076) as no background signal was noted yet positive controls provided maximal signal. Kinetic ELISAs were then performed as previously described to determine the Vmax in milli-units/min [3]. Antibody titers were defined as the inverse of the lowest dilution at specified Vmax threshold. We evaluated two thresholds, a Vmax of 25 and a Vmax of 50 to determine whether these correlated with protection against intestinal colonization in challenged mice (figure S1). Data between all methods was correlated using Spearman's correlation in SPSSv.27.

Figure S1.

	Colonization	IgG titer 25	IgG titer 50	IgG Avidity	IgA titer 25	IgA Titer 50	IgA Avidity	Steady State KD
**Colonization**		**0.271**	0.162	0.058	**0.256**	0.168	-0.082	-0.114
**IgG titer 25**	***0.005***		**0.928**	-0.129	**0.680**	**0.491**	-0.050	-0.153
**IgG titer 50**	0.137	***>0.001***		**-0.310**	**0.653**	**0.463**	0.109	-0.167
**IgG Avidity**	0.620	0.263	***0.016***		-0.057	0.030	0.261	-0.248
**IgA titer 25**	***0.013***	***>0.001***	***>0.001***	0.652		**0.875**	-0.200	0.394
**IgA Titer 50**	0.191	***>0.001***	***>0.001***	0.860	***>0.001***		**-0.615**	0.341
**IgA Avidity**	0.589	0.736	0.554	0.083	0.177	***0.009***		**-0.660**
**Steady State KD**	0.623	0.507	0.552	0.306	0.086	0.278	***0.005***	

Correlation Coefficient: 1 to -1

p-value: **0.05** to 1

Figure S1. *Protection against ETEC colonization is correlated with serum IgG and IgA responses.*

Titers were correlated using Spearman's correlation with 2-sided alpha of <0.05 considered significant. Correlation coefficients are shown in the upper right while *p*-values are listed in the bottom left. Significant values are in bold with the shading representing the strength of the correlation or significance.

At the lower threshold for positivity (Vmax of 25 milliunits/min) ELISA assays demonstrated strong correlation between IgG or IgA titers and colonization data in mice, while chaotropic avidity assays using 6M Urea were not associated with ETEC colonization. As ELISA assays offered a reproducible platform with good discriminatory power as well as a potential correlate of protection against colonization in the murine model, we adapted ELISA to the analysis of human serum samples from endemic areas.

Validation of dried blood spot preparation methodology.

Dried blood spots were reconstituted according to previously published work where reconstituted samples corresponded ~ to a 1:200 dilution of serum [4]. To validate this approach, we compared direct ELISA of hyperimmune rabbit sera previously raised against rEtpA[5] and rEatA passenger domain[6] to test samples spotted onto cards. Briefly, 1 ml of 3-4% RBCs was pelleted by centrifugation and resuspended in 100 µl of immune rabbit sera to approximate the concentration of RBCs in human blood. These resulting samples were spotted onto Whatman 903 Protein Saver cards, allowed to dry at room temperature, and stored in the dark for a minimum of 2 days prior to use. 3.2 mm punches were placed in microfuge tubes containing 300 µl PBS with 0.5% bovine serum albumin overnight, then centrifuged at 2,500 x g for 7 minutes and the supernatant removed. Reconstituted samples were stored at 4°C. Serial dilutions of immune rabbit sera and the reconstituted blood spots were then analyzed by ELISA (figure s2).

Figure S2

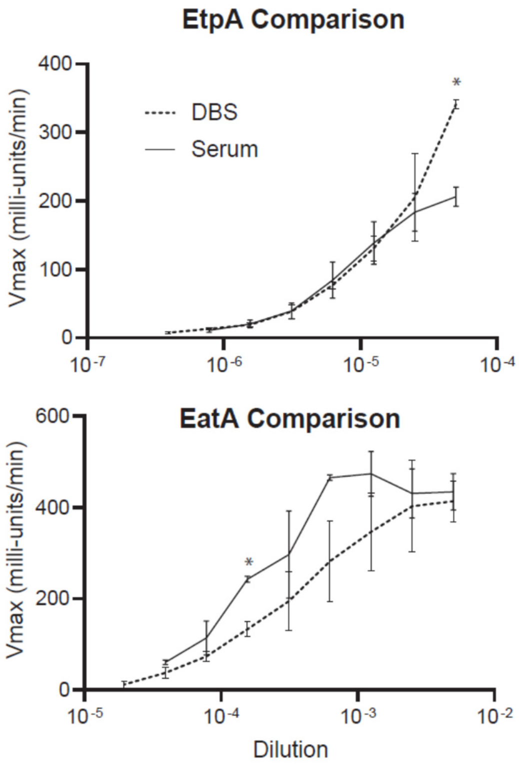

Figure S2. Comparison of Dried Blood Spots (DBS, dashed line) and sera (solid line) dilutions. The curves overlapped utilizing the 1:200 dilution previously reported (Intraclass Correlation Coefficient – 0.96 for the anti-EtpA ELISA and 0.92, anti-EatA).

Figure S3

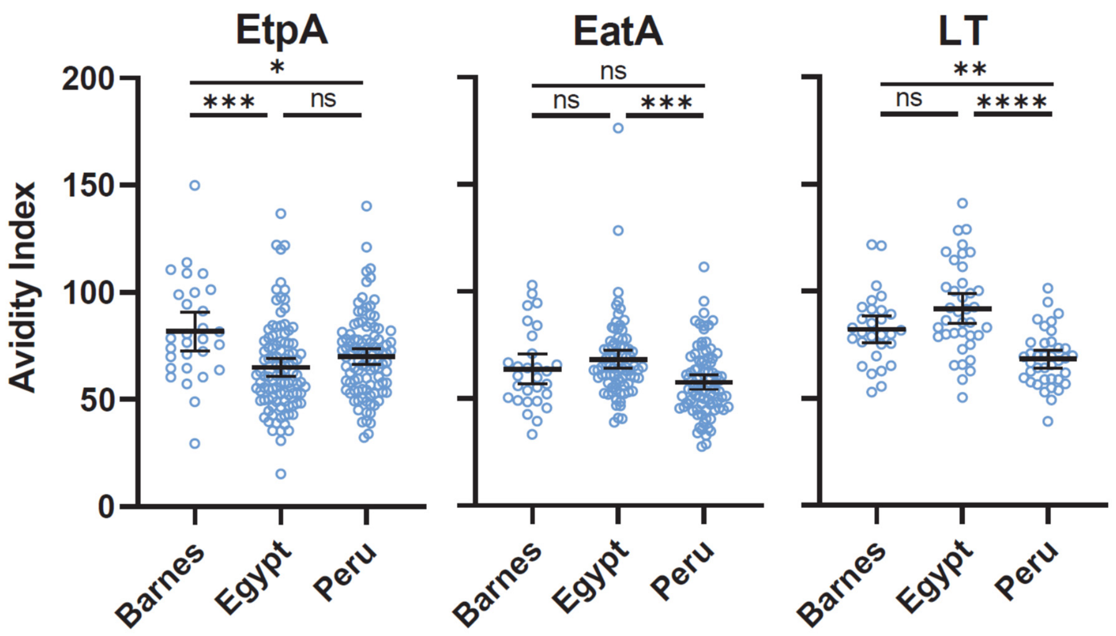

Figure S3. Avidity Index for adult IgA responses. Mean ± 95% Confidence Intervals are shown. Statistical differences were determined using ANOVA with Tukey's post-hoc analysis.

Table S1

**Table S1. bacterial strains and plasmids used in these studies**			
bacterial strains			
strain	description/genotype		reference(s)/ source
jf3227	*E. coli* Top10(pTV001)		
jf1696	*E. coli* Top10(pJL017/pJL030)		[5,7]
Top10	F-*mcrAΔ(mrr-hsdRMS-mcrBC) Φ80lacZΔM15 ΔlacX74 recA1 araD139 Δ(araleu)7697 galU galK rpsL* (StrR) *end*A1 *nup*G		Invitrogen
plasmids			
plasmid	description	addgene number	reference(s)
pTV001	EatA expression plasmid to produce polyhistidine-tagged EatA passenger domain with 8 x His at permissive site between amino acids 86-87 of EatA. Amp^R	130265	[8]
pJL017	EtpBA expression plasmid to produce polyhistidine-tagged EtpA. Amp^R	53532	[5,7]
pJL030	EtpC glycosyltransferase expression plasmid, Cm^R	53533	[5,7]

Table S2

Table S2a-f. Correlation of antibody responses within individuals at different sites.

S2a. Barnes (US Adults), ϱ(rho)* (*p*-value, N)

		LT		EtpA		EatA
		IgG	IgA	IgG	IgA	IgG
EatA	IgA	0.034 (0.862, 29)	0.116 (0.551, 29)	0.358 (0.056, 29)	***0.839 (0, 29)***	-0.038 (0.845, 29)
	IgG	0.106 (0.584, 29)	0.147 (0.447, 29)	-0.054 (0.782, 29)	-0.11 (0.571, 29)	
EtpA	IgA	0.132 (0.495, 29)	0.191 (0.321, 29)	0.345 (0.067, 29)		
	IgG	0.231 (0.229, 29)	0.039 (0.842, 29)			
LT	IgA	***0.45 (0.014, 29)***				

S2b. Egypt, ϱ(rho) (p-value, N)

		LT		EtpA		EatA
		IgG	IgA	IgG	IgA	IgG
EatA	IgA	-0.069 (0.489, 102)	***0.242 (0.014, 102)***	-0.026 (0.794, 102)	***0.769 (0, 102)***	0.031 (0.755, 102)
	IgG	0.186 (0.061, 102)	0.024 (0.809, 102)	***0.27 (0.006, 102)***	0.017 (0.865, 102)	
EtpA	IgA	-0.061 (0.545, 102)	***0.257 (0.009, 102)***	0.153 (0.124, 102)		
	IgG	***0.456 (0, 102)***	-0.039 (0.694, 102)			
LT	IgA	-0.046 (0.646, 102)				

S2c. Peru, ϱ(rho) (p-value, N)

		LT		EtpA		EatA
		IgG	IgA	IgG	IgA	IgG
EatA	IgA	0.053 (0.421, 231)	***0.372 (0, 231)***	***0.315 (0, 230)***	***0.583 (0, 231)***	***0.385 (0, 231)***
	IgG	***0.357 (0, 231)***	0.099 (0.132, 231)	***0.508 (0, 230)***	***0.256 (0, 231)***	
EtpA	IgA	-0.014 (0.837, 231)	***0.305 (0, 231)***	***0.322 (0, 230)***		
	IgG	0.053 (0.424, 230)	***0.261 (0, 230)***			
LT	IgA	0.094 (0.152, 231)				

S2d. Cameroon, ϱ(rho) (*p*-value, N)

		LT	EatA	EtpA
		IgG	IgG	IgA
EtpA	IgG	***0.549 (0, 167)***	***0.694 (0, 172)***	***0.503 (0, 141)***
	IgA	***0.49 (0, 136)***	***0.495 (0, 141)***	
EatA	IgG	***0.461 (0, 167)***		

S2e. SLCH (US Children), ϱ(rho) (p-value, N)

		LT		EtpA		EatA
		IgG	IgA	IgG	IgA	IgG
EatA	IgA	***0.342 (0.033, 39)***	***0.701 (0, 40)***	0.081 (0.644, 35)	***0.616 (0, 40)***	0.296 (0.064, 40)
	IgG	***0.368 (0.017, 42)***	0.301 (0.06, 40)	0.244 (0.141, 38)	***0.385 (0.014, 40)***	
EtpA	IgA	***0.481 (0.002, 39)***	***0.765 (0, 40)***	0.146 (0.402, 35)		
	IgG	***0.602 (0, 38)***	0.068 (0.697, 35)			

LT	IgA	***0.441 (0.005, 39)***

S2f. Haiti, ϱ(rho) (p-value, N)

		EtpA		EatA		LT
		IgA	IgG	IgG	IgA	IgG
LT	IgA	0.047 (0.587, 133)	0.093 (0.286, 133)	-0.069 (0.427, 133)	0.108 (0.217, 133)	***0.615 (0, 133)***
	IgG	0.116 (0.183, 133)	***0.226 (0.009, 133)***	0.106 (0.225, 133)	***0.222 (0.01, 133)***	
EatA	IgA	***0.734 (0, 133)***	***0.602 (0, 133)***	0.131 (0.133, 133)		
	IgG	0.119 (0.173, 133)	0.117 (0.178, 133)			
EtpA	IgG	***0.659 (0, 133)***				

*Pearson correlation

Table S3

Table S3. Correlation of antibody responses and age (N = 189)

		Correlation Coefficient*	p-value
EtpA	IgG	0.495	<0.001
	IgA	0.493	<0.001
EatA	IgG	0.323	<0.001
	IgA	0.446	<0.001
LT	IgG	0.321	<0.001
	IgA	0.098	0.179

**Pearson's correlation; log_{10} (Vmax) values were used to normalize the response distribution.*

Return to text

Table S4

Table S4. Negative correlation between Δ antibody response/one month[a] and age (N=125).

		Correlation Coefficient*	p-value
EtpA	IgG	-0.206	0.021
	IgA	-0.193	0.031
EatA	IgG	-0.236	0.008
	IgA	-0.173	0.054
LT	IgG	-0.209	0.019
	IgA	-0.089	0.322

[a] change in antibody response over the course of one month

*Pearson Correlation

SUPPLEMENTAL REFERENCES

1. Luo, Q.; Vickers, T.J.; Fleckenstein, J.M. Immunogenicity and Protective Efficacy against Enterotoxigenic Escherichia coli Colonization following Intradermal, Sublingual, or Oral Vaccination with EtpA Adhesin. *Clinical and vaccine immunology : CVI* **2016**, *23*, 628-637, doi:10.1128/CVI.00248-16.
2. Luo, Q.; Qadri, F.; Kansal, R.; Rasko, D.A.; Sheikh, A.; Fleckenstein, J.M. Conservation and immunogenicity of novel antigens in diverse isolates of enterotoxigenic Escherichia coli. *PLoS Negl Trop Dis* **2015**, *9*, e0003446, doi:10.1371/journal.pntd.0003446.
3. Tsang, V.C.; Wilson, B.C.; Maddison, S.E. Kinetic studies of a quantitative single-tube enzyme-linked immunosorbent assay. *Clinical chemistry* **1980**, *26*, 1255-1260.
4. Bhuiyan, M.S.; Hossain, M.; Sharmin, S.; Shirin, A.; Khanam, F.; Chowdhury, F.; Akter, A.; Khan, A.I.; Uddin, M.I.; Bhuiyan, T.R.; et al. Assessment of disease specific immune responses in enteric diseases using dried blood spot (DBS). *PLoS One* **2019**, *14*, e0218353, doi:10.1371/journal.pone.0218353.
5. Fleckenstein, J.M.; Roy, K.; Fischer, J.F.; Burkitt, M. Identification of a two-partner secretion locus of enterotoxigenic Escherichia coli. *Infection and immunity* **2006**, *74*, 2245-2258.
6. Patel, S.K.; Dotson, J.; Allen, K.P.; Fleckenstein, J.M. Identification and molecular characterization of EatA, an autotransporter protein of enterotoxigenic Escherichia coli. *Infection and immunity* **2004**, *72*, 1786-1794.
7. Fleckenstein, J.M.; Roy, K. Purification of recombinant high molecular weight two-partner secretion proteins from Escherichia coli. *Nat Protoc* **2009**, *4*, 1083-1092, doi:10.1038/nprot.2009.87.
8. Sheikh, A.; Wangdi, T.; Vickers, T.J.; Aaron, B.; Palmer, M.; Miller, M.J.; Kim, S.; Herring, C.; Simoes, R.; Crainic, J.A.; et al. Enterotoxigenic Escherichia coli Degrades the Host MUC2 Mucin Barrier To Facilitate Critical Pathogen-Enterocyte Interactions in Human Small Intestine. *Infection and immunity* **2022**, *90*, e0057221, doi:10.1128/IAI.00572-21.
